# Polarisationsabhängige Summenfrequenzspektroskopie (SFG) zur in situ Bestimmung der Nanopartikel‐Morphologie

**DOI:** 10.1002/ange.202300230

**Published:** 2023-04-04

**Authors:** Verena Pramhaas, Holger Unterhalt, Hans‐Joachim Freund, Günther Rupprechter

**Affiliations:** ^1^ Institut für Materialchemie TU Wien Getreidemarkt 9/BC 1060 Wien Österreich; ^2^ Fritz-Haber-Institut der Max-Planck-Gesellschaft Faradayweg 4–6 14196 Berlin Deutschland; ^3^ Derzeitige Adresse: ZKW Lichtsysteme Scheibbser Strassse 17 3250 Wieselburg Österreich; ^4^ Derzeitige Adresse: Robert Bosch GmbH Tübinger Straße 123 72762 Reutlingen Deutschland

**Keywords:** Metall Nanopartikel, Partikel Morphologie, Summenfrequenzspektroskopie, Vibrationsspektroskopie, in Situ Spektroskopie

## Abstract

Die Oberflächenstruktur von Metall‐Nanopartikel auf Oxidträgern lässt sich über charakteristische Schwingungen von adsorbierten Sondenmolekülen wie CO bestimmen. Üblicherweise konzentrieren sich spektroskopische Untersuchungen auf die Peak‐Position und ‐Intensität, die mit der Bindungsgeometrie bzw. der Anzahl der Adsorptionsplätze zusammenhängen. Anhand zweier unterschiedlich präparierter Modellkatalysatoren wird gezeigt, dass die polarisationsabhängige Summenfrequenzspektroskopie (SFG) die gemittelte Oberflächenstruktur und Form von Nanopartikel beleuchten kann. SFG‐Ergebnisse für verschiedene Partikelgrößen und Morphologien werden mit direkter Realraum‐Strukturanalyse mittels TEM und STM verglichen. Die beschriebene Anwendung von SFG kann zur in situ Detektion der Partikelstruktur verwendet werden und könnte ein wertvolles Werkzeug in der operando Katalyse werden.

## Graphical abstract

Moleküle auf geneigten Facetten: Polarisationsabhängige Summenfrequenzspektroskopie (SFG) von adsorbiertem CO wurde zur Charakterisierung der Morphologie von Pt und Pd Nanopartikel auf Oxidträgern eingesetzt. Das *I*_ssp_/*I*_ppp_ Verhältnis hängt von der Orientierung von CO relativ zur makroskopischen Oberflächennormalen ab und zeigt somit direkt den Beitrag geneigter/gekrümmter Facetten an.

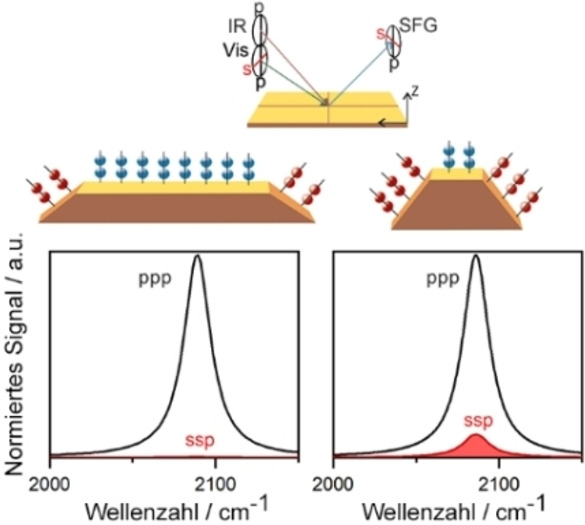

Die Summenfrequenzspektroskopie (SFG) ist eine vielseitig einsetzbare nichtlineare optische Schwingungsspektroskopie. Bei gleichzeitiger Anregung durch einen breitbandigen oder wellenlängendurchgestimmten mittleren Infrarot‐ (IR) und einen schmalbandigen sichtbaren (VIS) Laserpuls wird Licht mit der Summe der Einfallsfrequenzen erzeugt. Der zugrunde liegende Prozess ist nur für nicht‐zentrosymmetrische Medien (z. B. Oberflächen/Grenzflächen) erlaubt und weist somit eine inhärente Oberflächenempfindlichkeit auf.[^1^, ^2^] Im Gegensatz zu konventioneller IR‐Spektroskopie detektiert SFG daher nur Schwingungen von Molekülen, die an der Oberfläche adsorbiert sind, selbst wenn dieselben Moleküle auch in einer isotropen Gasphase vorhanden sind[^3^, ^4^, ^5^, ^6^, ^7^] (zur Unterscheidung von Oberflächen‐ und Gasphasenbeiträgen erfordert die IR‐Absorptionsspektroskopie Polarisations‐Modulation^[8]^). Dementsprechend kann SFG auch Moleküle an Luft‐Flüssig‐ und Flüssig‐Flüssig‐Grenzflächen und sogar “vergrabene” Grenzflächen innerhalb von Festkörpern charakterisieren.[^9^, ^10^, ^11^, ^12^] Darüber hinaus ermöglicht die starke Abhängigkeit des kohärenten SFG‐Lichts von der Orientierung und Häufigkeit der untersuchten Bindungen eine Analyse von Struktur und Oberflächenbedeckung, während die Orientierung molekularer Bindungen durch Verwendung verschiedener IR‐ und VIS‐Polarisationskombinationen bestimmt werden kann (basierend auf dem Tensorcharakter der nichtlinearen Suszeptibilität zweiter Ordnung).[^13^, ^14^, ^15^] SFG wurde in vielen Bereichen zur Untersuchung von Grenzflächenphänomenen eingesetzt, darunter Elektrochemie,^[16]^ Photokatalyse,^[17]^ Plasmonik,^[18]^ Polymere,^[19]^ Selbstorganisation^[20]^ und Nanomedizin.[^21^, ^22^]

In dieser Arbeit wurde polarisationsabhängige SFG angewendet, um molekulare Adsorption auf oxidgeträgerten Pt‐ und Pd‐Metall‐Nanopartikel (NP) zu untersuchen. Da das verwendete Sondenmolekül CO an den Top‐ und Seitenflächen von Pt‐ und Pd Partikel in gleicher Orientierung adsorbiert,[^13^, ^14^, ^15^, ^23^] wird bei SFG‐Messungen unterschiedlicher Polarisationskombinationen das Intensitätsverhältnis der Signale durch die Form der NP beeinflusst und nicht durch eine Änderung des Neigungswinkels der Adsorbate. Wir zeigen, dass das spektrale Intensitätsverhältnis die Gestalt von NP in zwei unterschiedlichen Modellkatalysatorsystemen widerspiegelt, was durch direkte morphologische Charakterisierung mittels Transmissionselektronenmikroskopie (TEM) und Rastertunnelmikroskopie (STM) bestätigt wird. Diese Methode ermöglicht die Verwendung von SFG zur in situ Evaluierung der mittleren NP‐Morphologie für Reaktionen, an denen CO ohnehin beteiligt ist oder die von CO‐Sondenmolekülen nicht negativ beeinflusst werden.^[24]^

Die Grundlagen der Summenfrequenzerzeugungsspektroskopie wurden in der Literatur ausführlich beschrieben.[^1^, ^2^, ^6^, ^8^, ^10^, ^11^, ^12^, ^13^] Die SFG‐Intensität *I*_SFG_ hängt linear von der Intensität der einfallenden Strahlen, *I*_IR_ und *I*_VIS_, und dem Betragsquadrat der nichtlinearen Suszeptibilität zweiter Ordnung *χ* ^(2)^ ab. Ein Schema der Strahlausbreitung und der möglichen Polarisationsrichtungen ist in Abbildung 1a dargestellt. Es wurde berichtet, dass auf Metallen nur zwei Polarisationskombinationen, ppp und ssp, ein signifikantes SFG‐Signal liefern:[^25^, ^26^]

(1) $$\chi_{eff,ppp}^{\left(2\right)}=\;-L_{xx}\left(\omega_{SFG}\right)L_{xx}\left(\omega_{Vis}\right)L_{zz}\left(\omega_{IR}\right)\cos\left(\alpha_{SFG}\right)\cos\left(\alpha_{Vis}\right)\sin\left(\alpha_{IR}\right)\chi_{xxz}-L_{xx}\left(\omega_{SFG}\right)L_{zz}\left(\omega_{Vis}\right)L_{xx}\left(\omega_{IR}\right)\cos\left(\alpha_{SFG}\right)\sin\left(\alpha_{Vis}\right)\cos\left(\alpha_{IR}\right)\chi_{xzx}+L_{zz}\left(\omega_{SFG}\right)L_{xx}\left(\omega_{Vis}\right)L_{xx}\left(\omega_{IR}\right)\sin\left(\alpha_{SFG}\right)\cos\left(\alpha_{Vis}\right)\cos\left(\alpha_{IR}\right)\chi_{zxx}+L_{zz}\left(\omega_{SFG}\right)L_{zz}\left(\omega_{Vis}\right)L_{zz}\left(\omega_{IR}\right)\sin\left(\alpha_{SFG}\right)\sin\left(\alpha_{Vis}\right)\sin\left(\alpha_{IR}\right)\chi_{zzz}$$

(2) $$\chi_{eff,ssp}^{\left(2\right)}=L_{yy}\left(\omega_{SFG}\right)L_{yy}\left(\omega_{Vis}\right)L_{zz}\left(\omega_{IR}\right)\sin\left(\alpha_{IR}\right)\chi_{yyz}$$

**Figure 1:**
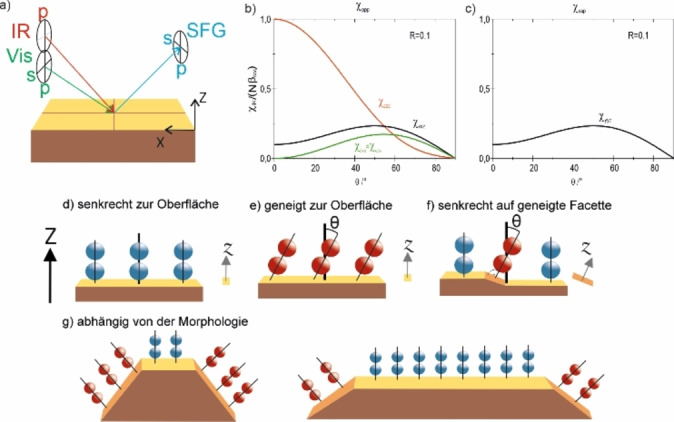
a) Mögliche Polarisationen beim SFG‐Prozess und Ausrichtung im Labor Referenzsystem. b), c) simulierte Beiträge zur effektiven nichtlinearen Suszeptibilität durch die nicht verschwindenden Elemente für ppp‐ und ssp‐Polarisationskombinationen, bei Verwendung eines niedrigen R=β_aac_/β_ccc_ von 0.1. d)–g) Einfluss geneigter Oberflächenfacetten auf die Bindungsorientierung von CO relativ zur makroskopischen Oberfächennormalen.

*L*_ab_ bezeichnet die Fresnel‐Faktoren und *χ_ijk_* bezeichnet die nicht verschwindenden Tensorelemente, wie sie in den Zu satzinformationen angegeben sind. *χ*_ppp_ ist eine lineare Kombination mehrerer Elemente (Abbildung 1b), gewichtet mit den Fresnel‐Faktoren. Für die aktuelle Messgeometrie hat sie ein Maximum bei einem Bindungsneigungswinkel von 0° und nimmt mit zunehmender Neigung monoton ab (mit *χ_zzz_* als Hauptbeitrag).^[13]^ Im Vergleich dazu ist *χ*_ssp_ direkt proportional zu *χ_yyz_*, sodass die ssp‐Intensität mit zunehmendem Neigungswinkel *θ* zwischen dem linearen CO‐Molekül und dem Normalvektor Z der Oberfläche zunimmt und ein Maximum bei etwa 45 bis 50° erreicht (siehe Abbildung 1c), was hauptsächlich von dem molekularen Tensorelementverhältnis R=β_aac_/β_ccc_ abhängt.[^13^, ^14^, ^15^]

Obwohl ein Vergleich von *I*_ppp_ und *I*_ssp_ erlaubt den Neigungswinkel von Molekülbindungen zu bestimmen, beinhalten SFG‐Untersuchungen von Metall‐Gas‐Grenzflächen meist nur ppp‐Spektren, da diese das stärkste Signal liefern. Ein Schlüsselaspekt der hier dokumentierten Beobachtungen ist schematisch in Abbildung 1d–f illustriert: Moleküle, die senkrecht auf einer planaren Oberfläche adsorbiert (blau) sind, weisen weder eine Neigung zur lokalen Oberflächennormalen z noch zur makroskopischen Normalen Z (Abbildung 1d) auf. Moleküle hingegen, die auf einer ebenen Fläche in geneigter Geometrie (rot) adsorbiert werden, sind sowohl in Bezug auf *z* wie *Z* geneigt (Abbildung 1e). Da SFG auf makroskopischer Ebene arbeitet, ist auch ein Molekül, das senkrecht auf einer geneigten Facette adsorbiert (parallel zu *z*), relativ zur makroskopischen Oberflächennormalen *Z* geneigt (Abbildung 1f). Daher trägt es wie ein Molekül mit geneigter Adsorptionsgeometrie zum Signal bei. Variationen der Partikelmorphologie, wie in Abbildung 1g dargestellt, führen daher bei SFG‐Messungen verschiedener Polarisationskombinationen zu unterschiedlichen Intensitätsverhältnissen, die die NP‐Form widerspiegeln.

In der vorliegenden Studie wurden zwei geträgerte Metall‐NP‐Systeme untersucht, Pt auf ZrO_2_ und Pd auf Al_2_O_3_. Die Messungen wurden in zwei ähnlichen Apparaturen durchgeführt, die jeweils aus einer Ultrahochvakuum (UHV)‐Oberflächenpräparations‐ und Analysekammer sowie einer damit verbundenen SFG‐Spektroskopiezelle für in situ Experimente von UHV bis Atmosphärendruck bestehen (ausführlich beschrieben in [27, 28]). Da die Metallnanopartikel >3.5 nm waren, sind keine Effekte durch das Trägermaterial zu erwarten.[^29^, ^30^]

Die Pt/ZrO_2_‐Modellkatalysatoren bestanden aus einem 42 nm dicken Zirkonia Film – gewachsen durch 400 Abscheidungszyklen (atomic layer deposition, ALD) auf einem Si(100)‐Wafer – und Pt‐Depositen, die durch eine unterschiedliche Anzahl von ALD‐Zyklen (10 bis 250) hergestellt wurden.^[33]^ TEM‐Bilder zeigten, dass 10 Pt Abscheidungszyklen zu Pt Partikel mit einer Größe von etwa 6 nm führen, während 250 Abscheidungszyklen einen homogenen Pt Film einer Dicke von etwa 10 nm erzeugen (Abbildung 2). Der Film schlägt eine Brücke zwischen früheren Einkristallstudien und den aktuellen NP‐Ergebnissen. Die Charakterisierung durch Elektronenmikroskopie, Röntgenbeugung und Photoelektronenspektroskopie ist in [33] beschrieben.

**Figure 2:**
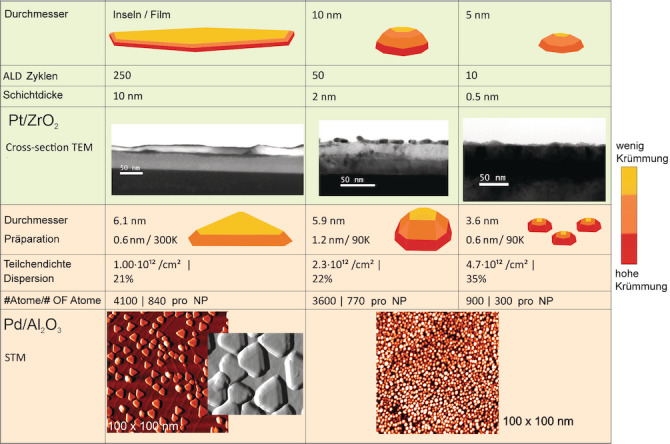
Morphologie von geträgerten Pt‐ und Pd‐Nanopartikel. Die durch ALD abgeschiedenen Pt Partikel wachsen anfänglich in Pyramidenform, die bei Erhöhung der Pt Menge runder wird. Die höchste Pt Exposition führt zu Koaleszenz, der Bildung von glatten Inseln und schließlich kontinuierlichen, glatten Filmen. Die Pd‐Partikelgröße wird durch die unterschiedliche Substrattemperatur und Pd‐Menge während PVD variiert. Bei 300 K wachsen Partikel in abgeschnittener kuboktaedrischer Form, mit einer großen, flachen (111) Top‐Facette. Bei 90 K werden rauere, halbkugelförmige NP gebildet, ebenso ist die Partikeldichte erhöht. STM‐Bilder mit Genehmigung adaptiert von Lit. [31] und [32].

Der zweite Probentyp, wohldefinierte Pd NP, wurde durch physikalische Gasphasenabscheidung (PVD) auf Aluminiumoxid‐Dünnschichten im UHV[^31^, ^32^, ^34^, ^35^, ^36^] gewachsen. Abbildung 2 zeigt STM‐Bilder von Pd NP, die auf Al_2_O_3_/NiAl(110) bei 300 K und 90 K Substrattemperatur gewachsen wurden. Bei 300 K gewachsene Pd NP sind abgeschnittene Kuboktaeder mit ausgeprägten, glatten (111) und (100) Facetten, während bei 90 K gewachsene Partikel eher rund/unregelmäßig erscheinen (d. h. die Facetten sind rauer mit mehr Stufen/Defekten). Für die im folgenden diskutierten Proben wurde jeweils eine nominale Dicke von 0.6 nm Pd bei 300 und 90 K abgeschieden, was gut facettierte Pd NP mit einer mittleren Größe von 6.1 nm bzw. rauere Pd NP mit einer mittleren Größe von 3.6 nm ergab. NP mit einer mittleren Größe von 5.9 nm wurden bei 90 K hergestellt, indem eine nominelle Dicke von 1.2 nm Pd abgeschieden wurde (für die Charakterisierung wurde hier Profilanalyse der Beugungsreflexe in niederenergetischer Elektronenbeugung (spot profile analysis of low energy electron diffraction, SPA‐LEED) vorgenommen^[35]^). Ähnlich wie bei den Pt Proben sind diese größeren Partikel höher als Partikel, die weniger Pd beinhalten, und haben daher ausgeprägtere seitlich gerichtete Facetten. Weitere Informationen lassen sich in den Zusatzinformationen finden.

Für beide Modellkatalysatorsysteme wurden durch polarisationsabhängige SFG‐Messungen von adsorbierten CO‐Molekülen drei wichtige Kenngrößen bestimmt: Die erste ist die Peakposition, die Informationen über die lokale Adsorptionsgeometrie (linear (on‐top), 2‐fach verbrückt (bridge), 3‐fach koordiniert (hollow)) liefert. Die exakte Resonanzposition, insbesondere für on‐top CO, wird außerdem durch die Oberflächenrauigkeit (Koordinationszahl des Metalls) und die CO‐Bedeckung (über Dipol‐Dipol‐Kopplung und chemische Verschiebung) beeinflusst.^[37]^ Die zweite Kenngröße ist die Intensität, die von der Häufigkeit der Adsorptionsplätze, der Bedeckung, der Ordnung und der Kopplung von Schwingungsmoden von adsorbiertem CO abhängt.[^7^, ^8^, ^37^] SFG‐Messungen unter Verwendung allein von ppp‐Polarisation zur Bestimmung der Größe von Pt^[38]^ und Pd Partikel^[39]^ sind in der Literatur zu finden. In diesen Studien wurde die Partikelstruktur identifiziert, indem die Peakpositionen und ‐intensitäten verglichen und verschiedene theoretische Simulationsmodelle als Referenz verwendet wurden. Die dritte Messgröße ist das Verhältnis von *I*_ssp_ zu *I*_ppp_, das die mittlere Orientierung der C−O‐Bindung in Bezug zur makroskopischen Oberflächennormalen widerspiegelt. Polarisationsabhängige (ssp und ppp) SFG‐Spektren von an Pt‐ und Pd‐Einkristallen adsorbiertem CO sind ebenso verfügbar[^8^, ^13^, ^14^, ^15^, ^40^] und dienen zum Benchmarking unserer Ergebnisse.

Abbildung 3 zeigt ppp‐ (in Schwarz) und ssp‐Spektren (in Rot) der ALD Pt Proben für (a) den kontinuierlichen Film, (b) abgerundete NP mit etwa 10 nm Durchmesser und (c) pyramidenförmige NP mit etwa 6 nm Durchmesser, alle gemessen in 10 mbar CO bei 425 K. Für den dünnen Pt‐Film wurde in ppp ein charakteristischer Peak von on‐top CO bei 2089 cm^−1^ beobachtet, der unter diesen Bedingungen gut mit 2090 cm^−1^ auf Pt(111) übereinstimmt^[41]^ (für Details zum Fit siehe Hintergrundinformationen). Wenn die Oberflächenrauigkeit zunimmt (d. h. die durchschnittliche Pt‐Koordinationszahl abnimmt), verschiebt sich der Peak über 2086 cm^−1^ auf 2057 cm^−1^. Die ssp‐Spektren spiegeln diesen Trend wider, trotz der leicht unterschiedlichen Phase des Signals. Für die pyramidenförmigen 6 nm Pt Partikel gibt es in ssp auch einen kleinen Peak bei 2086 cm^−1^, der auf einige (111) ähnliche Bereiche hinweist. Neben der Resonanzposition sind die *I*_ssp_/*I*_ppp_ Verhältnisse aufschlussreich. Die aus den gefitteten Spektren abgeleiteten Intensitätsverhältnisse, sowie Einkristall‐Referenzdaten, sind aus Tabelle 1 ersichtlich.[^13^, ^14^, ^15^] Glatte Pt‐Filme verfügen über keine oder nur wenige geneigte (Seiten‐)Facetten und haben damit ein kleines Verhältnis von Stufen‐ zu Terrassen‐Adsorptionsplätzen. Dementsprechend zeigen SFG‐Spektren des dünnen Pt Films ein starkes ppp‐Signal, aber ein sehr schwaches ssp‐Signal. Für die gekrümmten 10 nm Pt Partikel ist *I*_ssp_/*I*_ppp_ am größten (0.4), während es für die pyramidenförmigen 6 nm Pt Partikel aufgrund ihrer geneigten, aber weniger gekrümmten Oberflächen kleiner ist (0.3).

**Figure 3:**
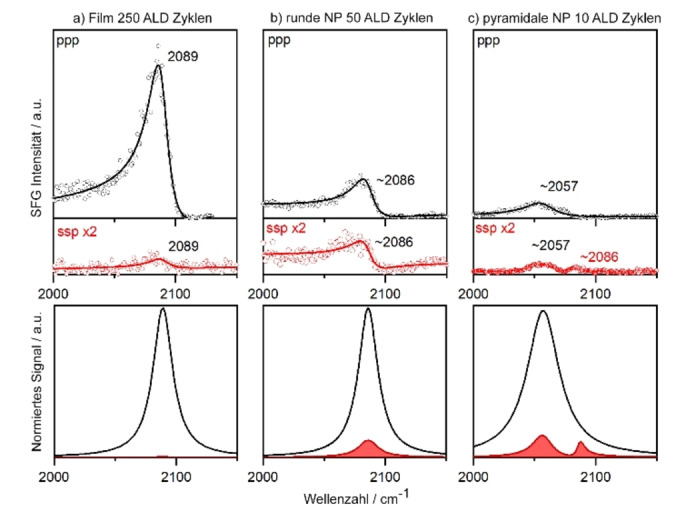
Polarisationsabhängige SFG‐Spektren von on‐top‐CO auf verschiedenen ALD‐gewachsenen Pt/ZrO_2_‐Katalysatoren. Die Messungen wurden in 10 mbar CO bei 425 K durchgeführt. Die schwarzen Spektren zeigen die ppp‐Polarisationskombination und verschieben sich für kleinere und rauere Pt Nanopartikel zu niedrigeren Wellenzahlen. Die roten Spektren zeigen die ssp‐Polarisationskombination, um den Faktor zwei gegenüber den ppp Spektren vergrößert. Die ssp‐Spektren sind für eine gekrümmte Partikelmorphologie vergleichsweise stärker. Die untere Reihe zeigt normierte spektrale Anpassungen (Fits) des resonanten Signals, in Abwesenheit des nicht‐resonanten Hintergrunds.

**Table 1:** *I*_ssp_/*I*_ppp_ Verhältnis (±10 %) für on‐top gebundenes CO auf verschiedenen Pt/ZrO_2_ Proben sowie verbrückt gebundenes CO auf den Pd/Al_2_O_3_ Katalysatoren.

Pt on‐top CO *I*_ssp_/*I*_ppp_			
Einkristall	Film	10 nm NPs	6 nm NPs
0.04^[13]^	0.05	0.40	0.30
Pd verbrücktes CO *I*_ssp_/*I*_ppp_			
Einkristall	6.1 nm NPs facettiert	5.9 nm NPs rau	3.6 nm NPs rau
0.02^[13]^ sim. 0.10[^14^, ^15^] exp.	0.20	0.30	0.49

Eine frühere polarisationsabhängige SFG‐Studie von Pt/SiO_2_ berichtete für 40 nm große polykristalline Partikel über eine signifikante Verstärkung der Intensitäten für beide Polarisationen aufgrund von Plasmonenresonanz.^[42]^ In unserer Studie haben wir diesen Effekt nicht beobachtet, was wahrscheinlich auf die geringere Größe und unterschiedliche Partikelform/‐struktur zurückzuführen ist.

Für PVD gewachsene Pd/Al_2_O_3_ Modellkatalysatoren wurden SFG‐Spektren von CO gemessen, adsorbiert an den Pd Partikel bei 200 K, nachdem die Oberfläche mit CO gesättigt wurde (Abkühlung von Raumtemperatur in 10^−6^ mbar CO). Die ppp‐ und ssp‐Polarisationskombinationen für gut facettierte Pd Partikel mit einem mittleren Durchmesser von 6.1 nm, sowie für rauere Pd Partikel mit 5.9 und 3.6 nm, sind in Abbildung 4 dargestellt. Im Vergleich zu den oben diskutierten Pt‐Spektren ist eine stark asymmetrische Linienform in ppp sichtbar, was mit dem NiAl‐Trägermaterial zu tun hat. Wie ausführlich in Referenz [43] berichtet, induziert NiAl einen viel stärkeren nicht‐resonanten Hintergrund, der wahrscheinlich von einem Interband‐Übergang im Substrat herrührt. Trotzdem liefert eine Spektrenanpassung die genauen Resonanzpositionen, normierte Fits sind in der unteren Reihe von Abbildung 4 ohne den nicht‐resonanten Hintergrund dargestellt. Für alle Pd NP Größen zeigten die ppp‐Spektren überwiegend brückengebundenes CO (bei 1981–1992 cm^−1^), das an Partikelkanten und Stufen adsorbiert ist.[^7^, ^8^, ^29^, ^34^, ^35^, ^36^] Dieser Peak wird durch Intensitätstransfer von verbrücktem CO auf den (111)‐ und (100)‐Flächen (Schulter bei 1960 cm^−1^) verstärkt. Die Spektren zeigten auch ein schwächeres on‐top CO bei 2085–2096 cm^−1^. Das Verhältnis von *I*_ssp_/*I*_ppp_ für brückengebundenes CO ist in Tabelle 1 zusammen mit Referenzdaten vom Pd(111)‐Einkristall angegeben, wohingegen die schwachen on‐top Peaks für Pd nicht berücksichtigt wurden.

**Figure 4:**
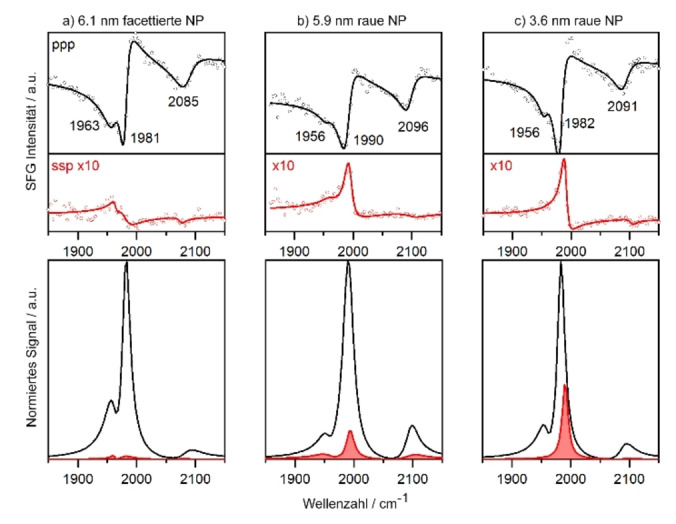
Polarisationsabhängige SFG‐Spektren von brückengebundenem und on‐top CO auf Pd/Al_2_O_3_ unterschiedlicher Partikelgröße und Rauigkeit. 6.1 nm Partikel wurden bei 300 K, 5.9 und 3.6 nm Partikel bei 90 K präpariert. Messungen wurden im UHV bei 200 K nach Sättigung der Oberfläche mit CO durchgeführt. Die roten Spektren zeigen die ssp‐Polarisationskombination, die um den Faktor zehn vergrößert wurde. Die ssp‐Spektren sind für gekrümmte Partikel vergleichsweise stärker. Die untere Reihe zeigt die normierten spektralen Fits des resonanten Signals ohne den nicht‐resonanten Hintergrund.

Wie oben beschrieben gibt es zwei unterschiedliche Formen/Morphologien der Pd Partikel. Die Partikelgestalt bestimmt das *I*_ssp_/*I*_ppp_ Verhältnis. Die bei 300 K gewachsenen 6.1 nm Pd Partikel sind gut facettierte, abgeschnittene Kuboktaeder[^31^, ^32^, ^34^, ^35^] mit einem Größe/Höhe‐Verhältnis von ungefähr 3. Die “großen” planaren (111) Deckfacetten führen zu einem starken ppp‐Signal von adsorbiertem CO, während das ssp‐Signal der kleineren, geneigten Seitenfacetten schwach ist. Dementsprechend ist *I*_ssp_/*I*_ppp_ ziemlich klein (0.2) und nähert sich dem von Pd(111) an (0.1). Die bei 90 K hergestellten 5.9 nm Pd NP haben einen ähnlichen mittleren Durchmesser, aber rauere und besser ausgeprägte Seitenfacetten. Als Ergebnis ist der Wert von *I*_ssp_/*I*_ppp_ von 0.3 höher. Im Vergleich dazu wurden die 3.6 nm großen Pd Partikel ebenfalls bei 90 K gewachsen und waren somit rau. Durch ihre noch kleinere Größe wird das *I*_ssp_/*I*_ppp_‐Intensitätsverhältnis sogar noch größer (0.49).

Zusammenfassend wurden polarisationsabhängige SFG‐Messungen für zwei Modellkatalysatorsysteme durchgeführt, bestehend aus unterschiedlichen Metall NP (Pt vs. Pd), abgeschieden mit verschiedenen Methoden (ALD vs. PVD), auf unterschiedlichen Trägermaterialien (ZrO_2_ vs. Al_2_O_3_), wobei CO bei unterschiedlichen CO Drücken (10 mbar vs. UHV) und unterschiedlichen Temperaturen (425 vs. 200 K) an verschiedenen Bindungsstellen (on‐top vs. verbrückt) bevorzugt adsorbierte. In beiden Fällen ergaben die polarisationsabhängigen SFG‐Messungen zusätzlich zu den typischerweise ausgewerteten Peakpositionen und ‐intensitäten *I*_ssp_/*I*_ppp_‐Verhältnisse, die die Partikelmorphologie/Oberflächenkrümmung im Einklang mit der mikroskopischen Charakterisierung widerspiegeln. Infolge dieser Übereinstimmung kann die polarisationsabhängige SFG‐Spektroskopie zur in situ Charakterisierung der Partikelmorphologie und insbesondere deren Änderungen eingesetzt werden, obwohl SFG normalerweise nicht zur Formcharakterisierung verwendet wird. Diese Morphologieanalyse unterscheidet sich von der Bestimmung der Oberflächenrauigkeit, die sich direkt aus der CO‐Resonanzposition und ihrer Verschiebung ergibt.

Als in situ spektroskopische Technik ermöglicht die polarisationsabhängige SFG insbesondere die Beobachtung von Veränderungen bei der Katalysator‐Aktivierung oder während katalytischer Reaktionen (Facettierung, Aufrauung, Sinterung etc.), während gleichzeitig die Reaktionsadsorbate und Zwischenprodukte überwacht werden können. Dies gilt für Reaktionen, an denen entweder CO beteiligt ist oder die durch adsorbiertes CO nicht beeinflusst werden. Die gezeigten “steady state” Spektren wurden unter stationären Bedingungen aufgenommen, aber kürzere Messzeiten könnten durch Begrenzung des Spektralbereichs, automatisches Umschalten von Polarisatoren oder sogar Breitband‐SFG erzielt werden. Die vorgestellte Methodik kann daher genutzt werden, die Morphologie von Modellkatalysator Nanoteilchen während der Herstellung, Vorbehandlung und katalytischer Reaktionen zu charakterisieren.

## Interessenkonflikt

Die Autoren erklären, dass keine Interessenkonflikte vorliegen.

## Supporting information

As a service to our authors and readers, this journal provides supporting information supplied by the authors. Such materials are peer reviewed and may be re‐organized for online delivery, but are not copy‐edited or typeset. Technical support issues arising from supporting information (other than missing files) should be addressed to the authors.

Supporting Information

## Data Availability

Die Daten, die die Ergebnisse dieser Studie unterstützen, sind auf begründete Anfrage beim Autor erhältlich.
