# Supplementary material for: Polarisationsabhängige Summenfrequenzspektroskopie (SFG) zur in situ Bestimmung der Nanopartikel‐Morphologie
Source: Angew Chem Weinheim Bergstr Ger. 2023 Apr 4;135(19):e202300230. doi: 10.1002/ange.202300230 (PMC10952846; doi:10.1002/ange.202300230)
Supplement: Supplementary file 1 — Supporting Information [file ANGE-135-0-s001.pdf]

Angewandte Chemie
Eine Zeitschrift der Gesellschaft Deutscher Chemiker

Supporting Information

Polarisationsabhängige Summenfrequenzspektroskopie (SFG) zur in situ Bestimmung der Nanopartikel-Morphologie

*V. Pramhaas, H. Unterhalt, H.-J. Freund, G. Rupprechter**

Supporting Information

1. SFG Theory

The basics of sum frequency generation (SFG) spectroscopy have been repeatedly described[1–3], with the SFG intensity I_{SFG} depending on the intensity of the incident beams, I_{IR} and I_{VIS}, and the second order nonlinear susceptibility $\chi^{(2)}$:

$$= I \quad \cdot I \qquad \cdot \left|\chi \quad ^{(2 \quad)}\right|^2,$$

with $\chi_{eff}^{(2)}$ around resonances modeled by

$$\chi_{eff}^{(2)} = \chi_{NR} + \sum_n \left(\frac{A_n}{\omega_{IR} - \omega_{nres} - \mathrm{i}\Gamma_n}\right),$$

where χ_{NR} is a contribution not influenced by the resonance, A_n is the Amplitude of the nth resonance, ω_{IR} is the IR frequency, ω_{nres} is the nth resonance frequency and Γ_n is the nth dampening coefficient connected to the excited state lifetime.

To calculate the tensor elements in laboratory frame, symmetry considerations on the molecular level are used. For a linear molecule like CO, with $C_{\infty v}$ symmetry, the largest component is β_{ccc}, while β_{aac} is small and all other tensor components of the molecular hyperpolarizability are zero[2].

<u>Linear optical Fresnel factors:</u>[3]

$$L_{xx}(\omega_\mathrm{i}) = \frac{2n_1(\omega_\mathrm{i})\cos\gamma}{n_1(\omega_\mathrm{i})\cos\gamma + n_2(\omega_\mathrm{i})\cos\beta}$$

$$L_{yy}(\omega_\mathrm{i}) = \frac{2n_1(\omega_\mathrm{i})\cos\beta}{n_1(\omega_\mathrm{i})\cos\beta + n_2(\omega_\mathrm{i})\cos\gamma}$$

$$L_{zz}(\omega_\mathrm{i}) = \frac{2n_2(\omega_\mathrm{i})\cos\beta}{n_1(\omega_\mathrm{i})\cos\gamma + n_2(\omega_\mathrm{i})\cos\beta}\left(\frac{n_1(\omega_\mathrm{i})}{n'(\omega_\mathrm{i})}\right)^2,$$

where β is the incidence angle and γ is the angle of refraction, n_1 and n_2 are the refractive indices of the bulk materials and n' is the refractive index of the interfacial layer.

Relevant non-vanishing Tensor elements:[2,4,5]

The non-zero tensor elements for the surface dependent susceptibility can then be calculated as

$$\chi_{zzz} = N\, \beta_{ccc}\, [R < cos\theta > + (1-R) < cos\theta >^3]$$

$$\chi_{yyz} = \chi_{xxz} = {}^1\!/_2\, N\, \beta_{ccc}[(1+R) < cos\theta > - (1-R) < cos\theta >^3]$$

$$\chi_{xzx} = \chi_{zxx} = \chi_{yzy} = \chi_{zyy} = {}^1\!/_2\, N\, \beta_{ccc}[(1-R) < cos\theta > - (1-R) < cos\theta >^3]$$

with $R = \frac{\beta_{aac}}{\beta_{ccc}}$, N the number of probed bonds and θ the tilt angle with respect to the surface normal. For more details on fitting the experimental spectra see refs. [5] and [6].

2. Sample Preparation

Pt particle preparation and characterization

The nucleation and growth of Pt particles is controlled by the surface temperature and cycle durations as well as the number of cycles in Atomic Layer Deposition (ALD).
Precursor temperature: 90 °C
1st precursor (MeCpPtMe$_3$) pulse time: 1s
2nd precursor (O_2) pulse time: 2s

Pt model catalysts were prepared in a separate ALD-chamber and transported through air to the SFG setup[6]. Therefore, samples were cleaned prior to use. As the standard cleaning procedure of single crystals in UHV (sputtering and annealing) would destroy the ALD prepared samples, they were pretreated by heating in 10 mbar O_2 to 400°C and in 20 mbar CO/O_2 (1:1) to 300°C, similar to (re-) activation of technological catalysts[7,8], yielding a CO covered surface. Thorough characterization of ALD model catalysts by scanning and transmission (cross-section) electron microscopy, X-ray diffraction and X-ray photoelectron spectroscopy was reported in the Supporting Information of ref [6].

Pd particle preparation and characterization

The alumina film (0.5 nm thick) was prepared by oxidation of a NiAl(110) alloy single crystal at 10^{-6} mbar O_2 at 543 K, followed by annealing at 1100 K. The Pd nanoparticles were grown on the oxide support by physical vapor deposition (PVD). The number density of Pd nanoparticles (particles per cm^2 of support) as well as their mean size (average number of atoms per particle) were controlled via the substrate temperature and the amount of evaporated metal (nominal film thickness monitored by a quartz microbalance), respectively. For more information refer to [9–14].

The number of Pd atoms per NP was derived from combined microbalance (number of deposited Pd atoms cm^{-2}) and scanning tunneling microscopy (STM; number of NPs cm^{-2}) measurements. STM was just used to provide particle dispersion and number, so that tip-convolution effects can be neglected. Assuming hemispherical shape, the average number of Pd atoms per nanoparticle can be converted to a mean nanoparticle size (with only small deviation for truncated half-octahedra). The STM-derived surface structures were further confirmed by infrared (IR) and SFG spectroscopy using CO as probe molecule[11–20]. For large Pd particles grown at 90 K the high particle density (number of particles per cm^2) hindered to exactly discriminate particles by STM. Instead, spot profile analysis of low energy electron diffraction (SPA-LEED) was used for characterization, as described in detail in ref. 10.

3. References

[1] Y. R. Shen, *The Principles of Nonlinear Optics*, Wiley-Interscience, Hoboken, N.J, **2003**.

[2] C. Hirose, N. Akamatsu, K. Domen, *J. Chem. Phys.* **1992**, *96*, 997–1004.

[3] X. Zhuang, P. B. Miranda, D. Kim, Y. R. Shen, *Phys. Rev. B* **1999**, *59*, 12632–12640.

[4] A. J. Moad, G. J. Simpson, *J. Phys. Chem. B* **2004**, *108*, 3548–3562.

[5] X. Li, V. Pramhaas, C. Rameshan, P. Blaha, G. Rupprechter, *J. Phys. Chem. C* **2020**, *124*, 18102–18111.

[6] V. Pramhaas, M. Roiaz, N. Bosio, M. Corva, C. Rameshan, E. Vesselli, H. Grönbeck, G. Rupprechter, *ACS Catal.* **2021**, *11*, 208–214.

[7] G. Rupprechter, G. Seeber, K. Hayek, H. Hofmeister, *Phys. Status Solidi A* **1994**, *146*, 449–459.

[8] G. Rupprechter, K. Hayek, H. Hofmeister, *J. Catal.* **1998**, *173*, 409–422.

[9] H.-J. Freund, *Angew. Chem.* **1997**, *109*, 444–468; *Angew. Chem. Int. Ed.* **1997**, *36*, 452-475.

[10] M. Bäumer, H.-J. Freund, *Prog. Surf. Sci.* **1999**, *61*, 127–198.

[11] M. Frank, M. Bäumer, *Phys. Chem. Chem. Phys.* **2000**, *2*, 3723–3737.

[12] H.-J. Freund, *Surf. Sci.* **2002**, *500*, 271–299.

[13] H.-J. Freund, M. Bäumer, J. Libuda, T. Risse, G. Rupprechter, S. Shaikhutdinov, *J. Catal.* **2003**, *216*, 223–235.

[14] A. Genest, J. Silvestre-Albero, W.-Q. Li, N. Rösch, G. Rupprechter, *Nat. Commun.* **2021**, *12*, 6098.

[15] K. Wolter, O. Seiferth, H. Kuhlenbeck, M. Bäumer, H.-J. Freund, *Surf. Sci.* **1998**, *399*, 190–198.

[16] T. Dellwig, G. Rupprechter, H. Unterhalt, H.-J. Freund, *Phys. Rev. Lett.* **2000**, *85*, 776–779.

[17] H. Unterhalt, G. Rupprechter, H.-J. Freund, *J. Phys. Chem. B* **2002**, *106*, 356–367.

[18] I. V. Yudanov, R. Sahnoun, K. M. Neyman, N. Rösch, J. Hoffmann, S. Schauermann, V. Johánek, H. Unterhalt, G. Rupprechter, J. Libuda, H.-J. Freund, *J. Phys. Chem. B* **2003**, *107*, 255–264.

[19] G. Rupprechter, in *Adv. Catal.,* Vol 51 (Eds.: B.C. Gates, H. Knözinger), Academic Press, **2007**, pp. 133–263.

[20] T. Lear, R. Marshall, J. Antonio Lopez-Sanchez, S. D. Jackson, T. M. Klapötke, M. Bäumer, G. Rupprechter, H.-J. Freund, D. Lennon, *J. Chem. Phys.* **2005**, *123*, 174706–174706.
